# Tunable VO_2_ cavity enables multispectral manipulation from visible to microwave frequencies

**DOI:** 10.1038/s41377-024-01400-w

**Published:** 2024-02-21

**Authors:** Hang Wei, Jinxin Gu, Tao Zhao, Zhiyuan Yan, He-Xiu Xu, Shuliang Dou, Cheng-Wei Qiu, Yao Li

**Affiliations:** 1 Center for Composite Materials and Structure, Harbin Institute of Technology, Harbin, 150001 China; 2 National University of Singapore, Department of Electrical & Computer Engineering, Singapore, 117583 Singapore; 3 School of Chemical Engineering and Technology, Harbin Institute of Technology, Harbin, 150001 China; 4 Suzhou Laboratory, Suzhou, 215123 China

**Keywords:** Nanocavities, Adaptive optics, Photonic devices

## Abstract

Optical materials capable of dynamically manipulating electromagnetic waves are an emerging field in memories, optical modulators, and thermal management. Recently, their multispectral design preliminarily attracts much attention, aiming to enhance their efficiency and integration of functionalities. However, the multispectral manipulation based on these materials is challenging due to their ubiquitous wavelength dependence restricting their capacity to narrow wavelengths. In this article, we cascade multiple tunable optical cavities with selective-transparent layers, enabling a universal approach to overcoming wavelength dependence and establishing a multispectral platform with highly integrated functions. Based on it, we demonstrate the multispectral (ranging from 400 nm to 3 cm), fast response speed (0.9 s), and reversible manipulation based on a typical phase change material, vanadium dioxide. Our platform involves tandem VO_2_-based Fabry–Pérot (F-P) cavities enabling the customization of optical responses at target bands independently. It can achieve broadband color-changing capacity in the visible region (a shift of ~60 nm in resonant wavelength) and is capable of freely switching between three typical optical models (transmittance, reflectance, and absorptance) in the infrared to microwave regions with drastic amplitude tunability exceeding 0.7. This work represents a state-of-art advance in multispectral optics and material science, providing a critical approach for expanding the multispectral manipulation ability of optical systems.

## Abstract (web summary)

We cascade VO_2_-based tunable optical cavities with selective-transparent layers to overcome the wavelength dependence, realizing the multispectral manipulation with reversible tunability covering wavelengths ranging from the VIS to MW regions.

## Graphical abstract

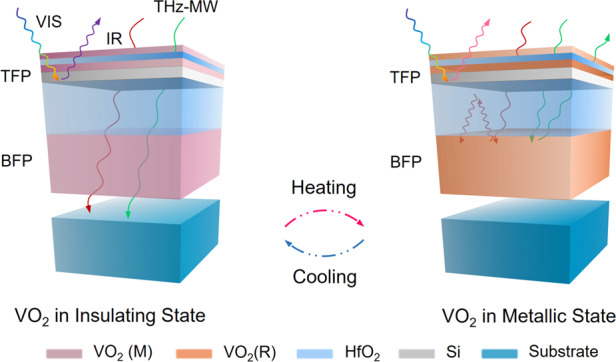

## Introduction

The demand for advanced applications in fields such as memories, information communication, imaging, and medical health has spurred the development of optical systems operating across broadband wavelengths ranging from the visible (VIS), infrared (IR) to terahertz (THz) and microwave (MW) regions^1–3^. For example, the development of materials with high IR emittance and solar reflectance can motivate terrestrial surfaces to cool themselves passively by harvesting the coldness of the universe^4–6^. However, optical systems with fixed spectra may generate undesired outcomes in non-standard conditions, such as overcooling^7,8^ and invalid concealment^9–13^, significantly limiting their practical applications.

Optical systems produced by phase change materials (PCMs)^14–17^ and electrochromic materials (ECMs)^18–20^ can overcome these challenges by dynamically changing optical responses under external stimulations. Overcooling can be prevented by automatically reducing the IR emittance when the temperature drops^7^. However, the strong and ubiquitous wavelength dependence observed in PCMs and ECMs restricts their unique optical properties to certain wavelengths^21^. Moreover, structures such as metasurfaces and optical cavities, which are deliberately designed to improve the efficiency of these materials, tend to exacerbate this limitation (Supplementary Fig. S1). As a result, optical systems that exhibit reversible tunability face severe challenges in achieving multispectral manipulation (Supplementary Table S1).

A significant breakthrough occurred when Kocabas et al.^21^ developed a multilayer graphene (MLG) based electrochemical optical platform capable of dynamically modulating reflectivity across a broad range of the swath from the VIS to MW regions. The MLG, which possesses high electrical conductivity (<50 Ω·sq^−1^), was laminated and vacuum sealed in a low-density polyethylene pouch. With optical transparency exceeding 90%, it can replace the top electrode and allows for broadband optical activity, opening up a new avenue for the development of ECMs-based optical systems. However, the ionic migration occurring inside ECMs inevitably leads to a relatively long response time in the range of dozens of seconds and additional energy consumption. The permanent tunable path from high absorption to high reflectance precludes possibilities of transmittance regulation. Despite the fair optical tunability of PCMs under external stimulations, their multispectral operations remain unreported due to the unbroken wavelength dependence (Supplementary Table S1).

In this article, we propose a universal method to breaking up the wavelength dependence of a typical PCM-vanadium dioxide (VO_2_), and demonstrate its multispectral manipulation with reversible tunability covering wavelengths ranging from the VIS to MW regions. The VO_2_-based system is capable of constructing a broadband color-changing space in the VIS region, and to the best of our knowledge, could switch freely between three typical optical models (transmittance, reflectance, and absorptance) in the IR to MW regions for the first time. Moreover, the ultrafast phase transition of VO_2_ enables faster response time of 0.9 s compared to ECMs-based systems. The unparalleled performance is unachievable for any reported optical system and represents a significant breakthrough in multispectral optics.

## Results

### Operating principle and ideal spectrum

**Fig. 1:**
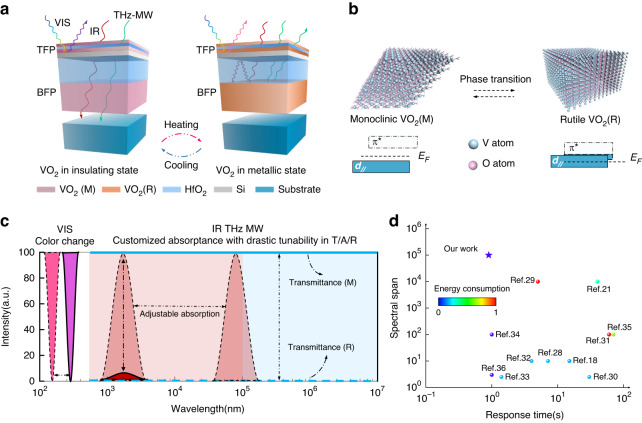
Schematic of proposed system with its operating principle. **a** Designed structures with VO_2_, HfO_2_, Si multilayers for reversible manipulation in the VIS, IR, THz and MW regions. From top to bottom, the thickness range for these layers are VO_2_ (500 nm)/HfO_2_ (depends on the requirement)/Si (150 nm)/VO_2_ (0–60 nm)/HfO_2_ (0–100 nm)/VO_2_ (0–20 nm); **b** Transformation of crystal and band structures during the phase transition of VO_2_; **c** The ideal spectrum of the proposed optical system ranging from VIS to MW regions, is drawn based on the measured spectra. The solid lines represent the spectra for VO_2_(M), and the dashed lines represent the spectra for VO_2_(R); It should be mentioned that the metallic VO_2_(R) induces the perfect absorption in IR, and the absorption wavelength can be dynamically modulated by structural parameters; **d** The comparison of covering bands, response time and energy consumption of different references. The spectral span is the ratio of the maximum and minimum wavelength of the dynamic control region^18,21,28–36^

Figure 1a demonstrates the proposed tandem Fabry–Pérot (F-P) cavity structure and its fundamental operating principle. At the critical temperature of 340 K, VO_2_ undergoes an ultrafast transformation from monoclinic VO_2_(M) to rutile VO_2_(R), accompanied by an energy band change that leads to the transition from insulating to metallic behaviors (Fig. 1b). The phase transition leads to high contrast in its optical behaviors from VIS to MW regions, thereby endowing VO_2_ with the potential for multispectral manipulation^22–27^. In the VIS region, the top VO_2_/HfO_2_/VO_2_/Si F-P cavity (TFP) enables the device to change reflective colors by adjusting resonant wavelengths during the phase transition. We choose Si, rather than the widely used Al, Ag or Au layers, as the bottom reflector in TFP to block VIS waves, because the transparency of Si in the IR to MW regions ensures the optical accessibility of the bottom cavity. Interestingly, TFP can be viewed as VO_2_/Si bi-layer due to the disappeared wavelength-dependent F-P effect when facing IR to MW waves, and determine the state of F-P resonance in the bottom F-P cavity (BFP) by the phase transition of VO_2_. Therefore, BFP can be regarded as a VO_2_/dielectric layer/VO_2_ tri-layer typical F-P structure, and is able to create a moveable F-P resonance peak in IR when VO_2_ is metallic. The bottom VO_2_ in BFP can block IR to MW waves due to the excellent conductivity of metallic VO_2_(R) while being transparent for insulating VO_2_(M). This is the key strategy to achieve comprehensive optical manipulation involving transmittance and meanwhile provide adequate reflectance in IR simultaneously. Figure 1c demonstrates the ideal multispectral manipulation in the proposed system based on the measured data: (i) broadband color-changing ability in VIS; (ii) drastic transmittance tunability from zero to near unity in the IR, THz and MW regions; (iii) A dynamic absorptance region whose peak position is also adjustable in IR and longer wavelengths; (iv) ultrafast switching speed. The experimental results demonstrate that our system is the only one reported so far, achieving multispectral and dynamic manipulation with ultrafast response speed based on PCM (Fig. 1d)^18,21,28–36^.

### Limitations of existing VO_2_-based systems in multispectral operations

**Fig. 2:**
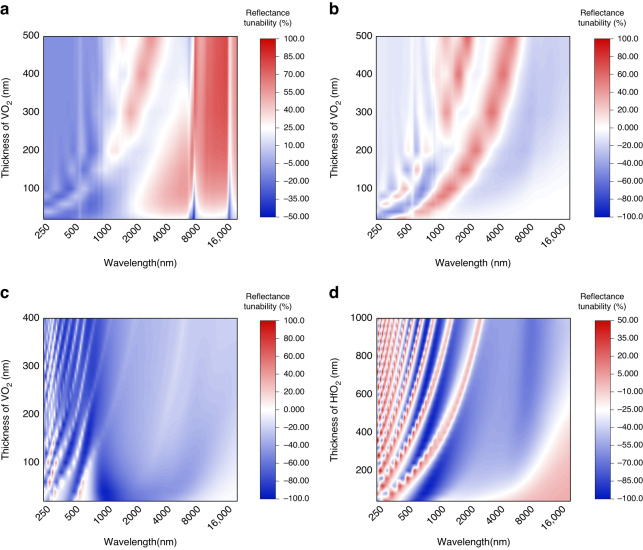
Demonstration of the wavelength dependence of common VO_2_-based optical systems. Simulated VIS-IR (250–25,000 nm) reflectance tunability of (**a**) VO_2_/SiO_2_; (**b**) VO_2_/Al; (**c**) VO_2_/HfO_2_/VO_2_ with varying VO_2_ thickness and (**d**) VO_2_/HfO_2_/VO_2_ with varying HfO_2_ thickness. We fix the HfO_2_ thickness to 200 nm in (**c**) and fix the VO_2_ thickness to 50 nm in (**d**)

The ubiquitous wavelength dependence of PCMs and ECMs confines their functionality to specific wavelengths^21^, whose modulation efficiency deteriorates seriously at other bands. We explore the wavelength dependence of VO_2_-based optical systems by the simulated thickness-dependent VIS-MIR reflectance tunability spectra of representative VO_2_-based platforms (VO_2_/SiO_2_, VO_2_/Al, and VO_2_/HfO_2_/Al). As shown in Fig. 2, it is apparent that there exists a certain region possessing the best modulation efficiency for fixed thickness. These regions shift to a longer wavelength with the increasing VO_2_ thickness and exhibit the typical sub-wavelength feature as a joint effect of the thickness-dependent interference effect^37^ and the intrinsic absorptance of VO_2_. Besides the strong wavelength dependence, the poor color-changing ability of VO_2_ in VIS and the shielding effect of common substrates (Al, Au, SiO_2_, et al.) in the IR to MW regions further limit the potential of VO_2_ for multispectral manipulation. Therefore, there are several issues in achieving the multispectral manipulation based on VO_2_: (i) improve the color-changing ability; (ii) overcome the wavelength dependence and (iii) break the shielding effect of substrates.

### Structural design for TFP operating in the VIS region

**Fig. 3:**
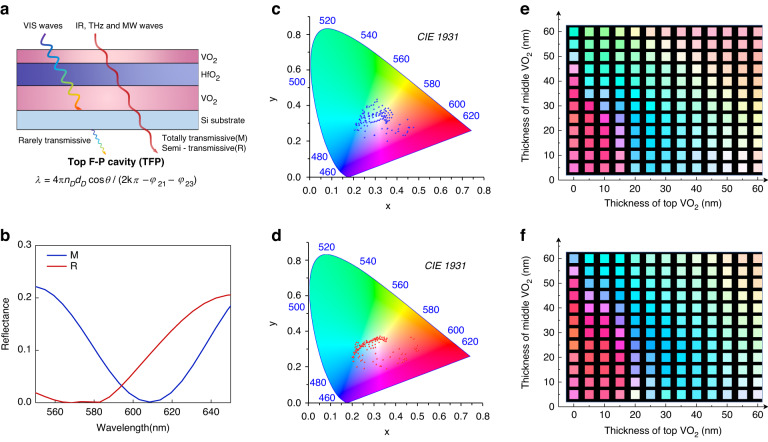
Structural design for TFP with the simulation in the VIS region. **a** Structural design for TFP with design principle; **b** Simulated VIS temperature-dependent reflectance spectra for 10 nm VO_2_/25 nm HfO_2_/40 nm VO_2_/150 nm Si. **c** Simulated surface colors in CIE 1931 space when VO_2_ is insulating; **d** Simulated surface colors in CIE 1931 space when VO_2_ is metallic; **e** Surface colors corresponding to specific thickness of VO_2_ layers when VO_2_ is insulating; **f** Surface colors corresponding to specific thickness of VO_2_ layers when VO_2_ is metallic

The slight permittivity discrepancy in the VIS region during the phase transition induces an inconspicuous color change of VO_2_^38^. (Supplementary Fig. S2a, b) Herein, as shown in Fig. 3a, we construct a TFP structure consisting of VO_2_/HfO_2_/VO_2_/Si layers to amplify the permittivity change of VO_2_ in the VIS region, and realize a reversible tuning of the reflective color. The top VO_2_ film acts as the semi-transparent layer and the bottom VO_2_ acts as a part of the dielectric layer. A 150-nm-thick Si layer is chosen as the substrate to block the propagation of VIS waves, while maintaining high transparency when facing IR to MW waves. (Fig. 3a and Supplementary Fig. S2c) The interactive disturbance in optical designs for TFP and BFP is simultaneously eliminated. Considering the coherent accumulation of the partial waves reflected from the F–P cavity^39,40^, the F-P resonant wavelength *λ* in TFP can be calculated as:

1 $${\rm{\lambda }}=4{\rm{\pi }}{n}_{D}{d}_{D}\cos \theta /(2k\pi -{\varphi }_{21}-{\varphi }_{23})$$

where *n*_*D*_ is the effective refractive index of dielectric layers consisting of HfO_2_ and VO_2_, *d*_*D*_ is the thickness of dielectric layers, *θ* is the incident angle, *φ*_*21*_ and *φ*_*23*_ represent the phase shift of the reflection coefficients at the top VO_2_/HfO_2_ interface and the VO_2_/Si interface. According to Eq. 1, ${n}_{D}$ is the primary factor contributing to tuning ${\rm{\lambda }}$ and changing surface colors during the phase transition. Meanwhile, different combinations of ${d}_{D}$, ${\varphi }_{21}$ and ${\varphi }_{23}$ enable the construction of a broadband color-changing gamut theoretically.

We then investigate the color-changing capacity of TFP by simulating the temperature-dependent reflectance spectra. For preset structural parameters (10 nm VO_2_/25 nm HfO_2_/40 nm VO_2_/150 nm Si), the amplified decrease in ${n}_{D}$ during the phase transition prompts the blue shift of the reflectance valley from 610 nm to 575 nm, with an encouraging color change observed from cyan to purple (Supplementary Fig. S2d and Fig. 3b). We further explore the potential of enriching the color-changing space by modifying structural parameters. Our calculations, presented in the CIE 1931 color space, demonstrate that it is achievable to gain diverse color-changing paths by varying the thickness of VO_2_ layers (Fig. 3c–f). TFP with a top-layer VO_2_ thinner than 20 nm exhibits superior dynamic color-changing capabilities across the phase transition. The ultra-thin thickness of each layer in TFP also ensures that it is optically accessible to BFP devices in the IR to MW regions. This section theoretically proves the capacity of TFP to generate broadband colors and determines an approximate thickness range for TFP^41^.

### Structural design for BFP operating in the IR to MW regions

Manipulating IR waves usually changes objects’ radiative properties, and further affects their heat exchange process. Thereby, the comprehensive capacity to switch between the three states of transmission, reflection, and absorption is crucial for a thermal regulator^42^. Constructing F-P cavities is the most common and efficient method to enhance the capacity of dynamic manipulating absorptance for VO_2_ in the IR to MW regions. However, as mentioned before, the strong wavelength dependence confines the spectral width, and the dynamic manipulation of transmittance is not supported as well.

**Fig. 4:**
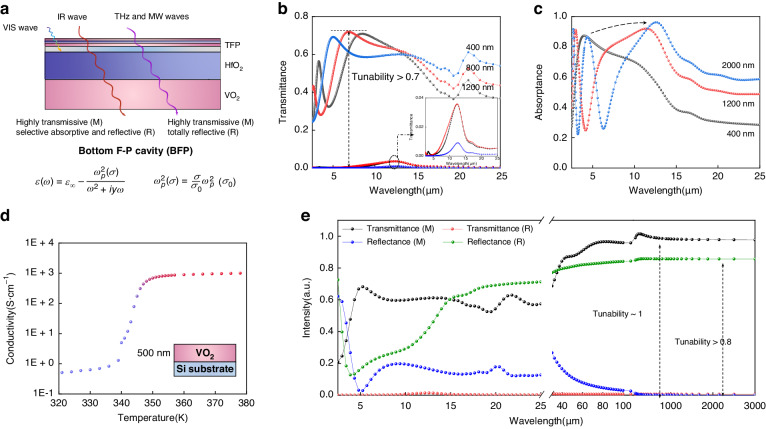
Structural design for BFP with the simulation in the IR to MW regions. **a** Structural design for BFP with design principle; **b** Simulated temperature-dependent IR transmittance spectra for different HfO_2_ thickness; **c** Simulated IR absorptance spectra for different HfO_2_ thickness when VO_2_ is metallic; **d** Measured temperature-dependent conductivity of 500 nm VO_2_ film deposited on a Si substrate; **e** Simulated temperature-dependent transmittance/reflectance spectra in IR to MW regions of BFP. The thickness of HfO_2_ in BFP is set to 400 nm

Herein, we propose a “two birds with one stone” method that replaces the metal reflector with a VO_2_ layer, whose reflection to IR, THz and MW waves is activated in its metallic state, and therefore achieves the broadband manipulation of transmittance while maintaining the resonant absorption in an F-P cavity (Fig. 4a). Simulated spectra in Supplementary Fig. S3 find that the minimum thickness for a VO_2_ layer to provide adequate reflectance in the IR region is around 300 nm. In this article, we choose a 500 nm thickness VO_2_ layer as the reflector for simulations and experiments.

Then we investigate the effect of structural parameters in TFP on the performance of BFP. As shown in Supplementary Fig. S4, the small deviations in spectra between the two structures reveal that it is acceptable to regard the VO_2_/HfO_2_/VO_2_ tri-layers in TFP as a monolayer VO_2_ layer whose thickness is the sum of the thickness of two VO_2_ layers. Supplementary Fig. S5 indicates that arbitrary thickness combinations of the two VO_2_ layers in TFP have no impact on the IR performance when maintaining the sum of their thickness to be a constant, but meanwhile, the system could exhibit diverse surface color appearances. It represents the achievable independent spectral manipulation in divided wavelengths. Meanwhile, we discover the effect of HfO_2_ thickness (varies from 0 to 2000 nm) on the IR performance of BFP and the comprehensive modulation paths covering the transmittance, reflectance and absorptance (Supplementary Fig. S6 and Fig. 4b, c). When VO_2_ is insulating, the system exhibits a maximum transmittance exceeding 0.7 because of the negligible optical loss in each layer. The reflectance at regions shorter than 5 μm increases slightly as a result of the refractive mismatch in interfaces within TFP, which indicates the sub-wavelength, or wavelength-dependence effect of optical systems. After the phase transition, the completely forbidden transmission in the IR region leads to a drastic transmittance tunability of 0.7. The F-P resonance generates a strong absorption peak whose position is adjustable by varying the thickness of HfO_2_ in BFP. In the non-resonant regions, the VO_2_(R) reflector enhances the reflectance significantly with maximum tunability exceeding 0.7. For potential applications requiring not only tunable amplitude but a moveable absorptance peak wavelength, it is also feasible by replacing the HfO_2_ layer with dielectric materials with changeable refractive index, such as liquid crystals^43^, GST alloys^44^.

In the THz and MW regions, the dielectric constants of VO_2_ can be described using the Drude model^45,46^:

2 $${\rm{\varepsilon }}\left({\rm{\omega }}\right)={\varepsilon }_{\infty }-\frac{{\omega }_{p}^{2}\left(\sigma \right)}{{\omega }^{2}+i\gamma \omega }$$

3 $${\omega }_{p}^{2}\left(\sigma \right)=\frac{\sigma }{{\sigma }_{0}}{\omega }_{p}^{2}\left({\sigma }_{0}\right)$$

where *ε*_∞_ = 12 is dielectric permittivity at the infinite frequency, ${\omega }_{p}(\sigma )$ is the plasma frequency of VO_2_ that dependent on conductivity,$\gamma$ = 5.75 ×10^13 ^rad· s^−1^ is the collision frequency, ${\sigma }_{0}$ = 3 ×10^5 ^S· m^−1^,$\,{\omega }_{p}({\sigma }_{0})$ = 1.5 ×10^15 ^rad· s^−1^, $\sigma$ is the conductivity of VO_2_ (Fig. 4d and Supplementary Fig. S7). As shown in Fig. 4e, when VO_2_ is insulating, BFP exhibits a completely transmissive state due to the lossless layers and the disappearance of interlayer interaction due to the wavelength dependence. After the phase transition, the transmittance drastically becomes 0, where the reflectance reaches 0.8 simultaneously. The performance may contribute to the combined applications of communications, computations and electromagnetic shielding^47,48^. In addition, systems deposited on substrates with different optical properties can bestow more manipulation paths in the IR to MW regions. Supplementary Fig. S8 demonstrates the simulated IR to MW spectra of systems deposited on totally absorptive/reflective substrates (Fig. 4e can be regarded as the spectrum of the system deposited on a transparent substrate). The radically different features in these spectra will activate and support much more insights into expansive multispectral applications.

### Experimental demonstration and characterization

**Fig. 5:**
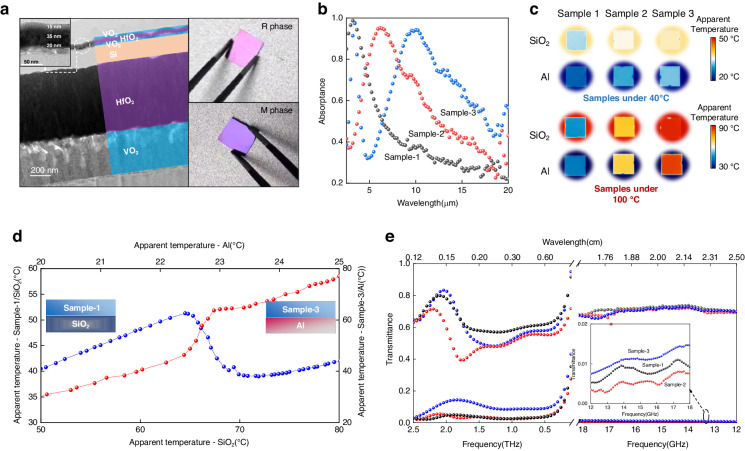
Experimental demonstration of proposed multispectral system. **a** Cross-section HRTEM image of Sample-2 with its color-changing capacity recorded by a camera; **b** The absorptance spectra of PoC samples when VO_2_ is metallic; **c** IR images taken by an IR camera when put PoC samples on Al and SiO_2_ wafers at 40 °C and 100 °C; **d** Distinct temperature-dependent absorptance paths for Sample-1 and Sample-3 put on SiO_2_ and Al wafers; **e** Measured temperature-dependent THz to MW transmittance spectra of three samples. The bottom lines near to zero represent the spectra measured at 100 °C, and top lines reaching 0.7 represent the spectra measured at room temperature

To further validate the promise of multispectral dynamic manipulation, we develop proof of concept (PoC) samples. (see Methods for details) A 300 μm thickness Si wafer with high transparency in the IR, THz and MW regions is chosen as the substrate to discover the consistency between experiments and simulations. The membrane thicknesses in TFP, from top to bottom, are 15, 35, 20, and 150 nm. The samples are labeled Sample-1, Sample-2, and Sample-3, based on the different thicknesses (0, 600, and 1200 nm) of the HfO_2_ layer in BFP. The thickness of the bottom VO_2_ in BFP maintains 500 nm. (Fig. 5a and Supplementary Fig. S9) Measured physical properties of the deposited VO_2_ films are presented in Supplementary Fig. S10.

In the VIS region, an apparent reflective color change from purple to pink is observed in Fig. 5a and Supplementary Movie 1 when heating the samples to 100 °C. The color change path meets well with the simulation in Fig. 2e, f. The blue shift of the F-P resonance in TFP during the phase transition is also detected in the measured temperature-dependent reflectance spectra, though errors of optical constants induce some mismatch in the intensity compared to the simulated spectra (Supplementary Fig. S11). Therefore, it is achievable to generate arbitrary colors by simply altering the layer thickness in TFP, which expands the potential of VO_2_-based optical systems in the fields of color display and adaptive camouflage. Meanwhile, the response time can be ascertained by measuring the time interval between the sample’s contact with the heater and its full discoloration, which occurs in approximately 0.9 s (Supplementary Fig. S12).

In the IR region, a measured maximum transmittance difference of 0.6 across the phase transition of VO_2_ in Supplementary Fig. S13a indicates the success in introducing the reversible tunability of transmittance into a multispectral optical system. The system exhibits an anti-reflection effect on the Si substrate so that the transmittance at around 7 μm is even higher than that of the substrate. The decreased transmittance in longer wavelengths due to the increased absorption of thicker HfO_2_ can be addressed by replacing the HfO_2_ dielectric layer with an IR lossless material. A large reflectance tunability of around 0.5 is also presented in Supplementary Fig. S13b. We further investigate the adjustable absorption performance of the samples. Figure 5b shows near-perfect absorption peaks generated by the F-P resonance in BFP when VO_2_ is metallic in all samples, which indicates a high absorptance tunability reaching 0.7 at resonant wavelengths, and the position can be easily adjusted within a wide range (3-11 μm) even extended to the THz and MW regions^49,50^ (Supplementary Fig. S13c). In addition, we further verify the effect of substrates on the dynamic manipulation path in the IR region. We put three samples on substrates with high emittance (SiO_2_ wafer) and high reflectance (Al wafer), then record the temperature-dependent IR images and videos (Fig. 5c and Supplementary Movies 2-3). The high transparency makes samples’ IR performance depend on the substrate at low temperatures, while eventually coming to a substrate-independent high absorptance state after the VO_2_ phase transition. Therefore, it is achievable to construct two distinctly opposite temperature-dependent absorptance tunability paths in the IR region, which can be adapted to requirements of different IR applications (Fig. 5d).

In the THz and MW regions, a sharp change of conductivity with more than three orders of magnitude is observed for a 500 nm VO_2_ layer deposited on a Si substrate (Fig. 4d). Measured temperature-dependent transmittance spectra in the THz and MW regions exhibit drastic transmittance tunability exceeding 0.7 for all samples (Fig. 5e). The wavelength dependence keeps THz and MW spectra unchangeable with varying HfO_2_ thicknesses. The absorptance of the Si substrate triggers a slight degeneracy in transmittance when VO_2_ is insulating compared to simulations.

Finally, response time, as another critical performance of multispectral manipulation, is also crucial^21^. The ultrafast phase transition of VO_2_ under the picosecond scale endows this system with an incomparable response time and has been widely confirmed^51^. Therefore, our work reports an unparalleled performance in multispectral manipulation (Fig. 1d and Supplementary Table S1).

## Discussion

In summary, we propose a universal method for overcoming the wavelength dependence of tunable optical materials, and have demonstrated the exceptional potential of modified tandem VO_2_-based F-P cavities for multispectral and reversible manipulation spanning from the VIS to MW regions. The multispectral platform achieves unprecedented performance, including broadband color-changing capability in the VIS region and free switching ability among three optical states of transmittance, reflectance, and absorptance in the IR to MW regions. Moreover, the ultrafast phase transition of VO_2_ enables a short response time of less than 0.9 s. Our work represents the state-of-the-art level in interdisciplinary research that interfaces multispectral optics and material science. We believe it will open up a wide range of possibilities for advanced applications in memories, thermal management, imaging, and communications.

## Methods

### Simulation method

Electromagnetic wave calculations are performed using FDTD Solutions, a commercially available FDTD simulation software from Lumerical Solutions, Canada. All of the simulations reported in this paper are performed in three-dimensional layouts. We use the periodic boundary conditions along the x and y axes, and use perfectly matched layers along the z axis. Mesh accuracy is set to 8, and the minimum mesh step is 0.00025 μm. The minimum auto shutoff is 10^−5^. A plane wave is chosen as the source which injects along the z axis. The transmittance and reflectance are monitored by power monitors. Optical constants of used materials at different regions can be found in the reference^52^. The physical optical models for VO_2_ at metallic and insulating states can be described as Eq. (4)^53^

4 $${\rm{\varepsilon }}\left({\rm{\omega }}\right)={{\rm{\varepsilon }}}_{\infty }+\frac{\left({{\rm{\varepsilon }}}_{{\rm{s}}}-{\rm{\varepsilon }}\right)\cdot {{{\rm{\omega }}}_{{\rm{t}}}}^{2}}{{{{\rm{\omega }}}_{{\rm{t}}}}^{2}-{{\rm{\omega }}}^{2}+{\rm{i}}{\Gamma }_{0}\cdot {\rm{\omega }}}+\mathop{\sum }\nolimits_{{\rm{j}}=1}^{\rm{n}}\frac{{{\rm{f}}}_{{\rm{j}}\cdot {{{\rm{\omega }}}_{0{\rm{j}}}}^{2}}}{{{{\rm{\omega }}}_{0{\rm{j}}}}^{2}-{{\rm{\omega }}}^{2}+{\rm{i}}{{\rm{\gamma }}}_{{\rm{i}}}\cdot {\rm{\omega }}}+\frac{{{{\rm{\omega }}}_{{\rm{p}}}}^{2}}{-{\rm{\omega }}+{\rm{i}}{\Gamma }_{{\rm{d}}}\cdot {\rm{\omega }}}$$

### VO_2_ layer deposition

VO_2_ layers were deposited by a high-power pulsed magnetron sputtering system (MS650C) purchased from KeYou, China. During the deposition process, the Ar/O_2_ ratio was fixed at 81:1.9 sccm, and the sputtering power was set to 180 W. The chamber pressure, substrate temperature, input pulse frequency and width were fixed at 0.9 Pa, 550 °C, 200 Hz and 50 μs, respectively.

### HfO_2_ layer deposition

HfO_2_ layers were deposited by a direct current magnetron sputtering system (MS650C) purchased from KeYou, China. During the deposition process, the Ar/O_2_ ratios were fixed at 81:6.8 sccm, and the sputtering powers were set to 220 W. The chamber pressure and the substrate temperature were fixed at 0.9 Pa, 400 °C, respectively.

### Si layer deposition

The Si layer was deposited by a radio frequency magnetron sputtering system (MS650C) purchased from KeYou, China. During the deposition process, the Ar/O_2_ ratios were fixed at 81:0 sccm, and the sputtering powers were set to 100 W. The chamber pressure and the substrate temperature were fixed at 0.9 Pa, 100 °C, respectively.

### Measurements

The crystalline phases were characterized using X-ray diffraction (XRD, PANalytical B.V. Model Xpert Pro). The Raman spectrum was recorded by an inVia Laser Micro Raman spectrometer (Renishaw, UK) equipped with a confocal microscope and a 532 nm excitation laser source. The cross-sectional images were evaluated by a field emission transmission electron microscope (JEOL JEM-F200). TEM samples were prepared by a focused ion beam (FIB) instrument (Hitachi NX5000). Fourier transform infrared (FT-IR) spectroscopy was performed using an FT-IR system (VERTEX-70, Bruker) from 2.5 to 25 μm. X-ray photoelectron spectroscopy (XPS) was performed with a PHI 5700 ESCA System using Al Ka radiation (1486.6 eV). XPS data were calibrated to the C1s peak and analyzed using Casa XPS software. The IR images were recorded by thermal imager (TIX-660, FLUKE) from 7.5 to 14 μm. The conductivity was measured by Hall Effect Measurement System (Ecopia HMS-3000).

### Supplementary information

Supplementary Materials

Supplementary Movie.1

Supplementary Movie.2

Supplementary Movie.3

## References

[1] Li Y (2021). Transforming heat transfer with thermal metamaterials and devices. Nat. Rev. Mater..

[2] Liu TJ (2022). Thermal photonics with broken symmetries. eLight.

[3] Huang G (2022). Upconversion nanoparticles for super-resolution quantification of single small extracellular vesicles. eLight.

[4] Yin XB (2020). Terrestrial radiative cooling: Using the cold universe as a renewable and sustainable energy source. Science.

[5] Li T (2019). A radiative cooling structural material. Science.

[6] Zeng SN (2021). Hierarchical-morphology metafabric for scalable passive daytime radiative cooling. Science.

[7] Tang KC (2021). Temperature-adaptive radiative coating for all-season household thermal regulation. Science.

[8] Wang SC (2021). Thermochromic smart windows with highly regulated radiative cooling and solar transmission. Nano Energy.

[9] Hu R (2021). Thermal camouflaging metamaterials. Mater. Today.

[10] Tang KC (2020). A thermal radiation modulation platform by emissivity engineering with graded metal–insulator transition. Adv. Mater..

[11] Qin B (2023). Whole-infrared-band camouflage with dual-band radiative heat dissipation. Light Sci. Appl..

[12] Xu ZQ (2020). Spatially resolved dynamically reconfigurable multilevel control of thermal emission. Laser Photonics Rev..

[13] Hou JF, Situ G (2022). Image encryption using spatial nonlinear optics. eLight.

[14] Abdollahramezani S (2022). Electrically driven reprogrammable phase-change metasurface reaching 80% efficiency. Nat. Commun..

[15] Wei H (2021). Smart materials for dynamic thermal radiation regulation. Small.

[16] Dou SL (2021). Bioinspired microstructured materials for optical and thermal regulation. Adv. Mater..

[17] Shahsafi A (2019). Temperature-independent thermal radiation. Proc. Natl Acad. Sci. USA.

[18] Li MY (2020). Manipulating metals for adaptive thermal camouflage. Sci. Adv..

[19] Ergoktas MS (2020). Graphene-enabled adaptive infrared textiles. Nano Lett..

[20] Strand MT (2021). Polymer inhibitors enable >900 cm^2^ dynamic windows based on reversible metal electrodeposition with high solar modulation. Nat. Energy.

[21] Ergoktas MS (2021). Multispectral graphene-based electro-optical surfaces with reversible tunability from visible to microwave wavelengths. Nat. Photonics.

[22] Cui YY (2018). Thermochromic VO_2_ for energy-efficient smart windows. Joule.

[23] Shi R (2019). Recent advances in fabrication strategies, phase transition modulation, and advanced applications of vanadium dioxide. Appl. Phys. Rev..

[24] Lai WE (2021). Fully optically tunable and flexible composite films for enhanced terahertz control and multifunctional terahertz devices. ACS Appl. Electron. Mater..

[25] Shi R (2023). Liquid precursor-guided phase engineering of single-crystal VO_2_ beams. Angew. Chem. Int. Ed..

[26] Shi R (2021). Phase management in single-crystalline vanadium dioxide beams. Nat. Commun..

[27] Chen BW (2023). Directional terahertz holography with thermally active Janus metasurface. Light Sci. Appl..

[28] Shao ZW (2022). All-solid-state proton-based tandem structures for fast-switching electrochromic devices. Nat. Electron..

[29] Yuan LM (2022). A dynamic thermal camouflage metadevice with microwave scattering reduction. Adv. Sci..

[30] Li WJ (2020). Effect of independently controllable electrolyte ion content on the performance of all-solid-state electrochromic devices. Chem. Eng. J..

[31] Mandal J (2018). Li_4_Ti_5_O_12_: a visible-to-infrared broadband electrochromic material for optical and thermal management. Adv. Funct. Mater..

[32] Zhang X (2019). Preparation and performances of all-solid-state variable infrared emittance devices based on amorphous and crystalline WO_3_ electrochromic thin films. Sol. Energy Mater. Sol. Cells.

[33] Zhao YM (2021). Preparation of Sn-NiO films and all-solid-state devices with enhanced electrochromic properties by magnetron sputtering method. Electrochim. Acta.

[34] Wang SC (2021). Scalable thermochromic smart windows with passive radiative cooling regulation. Science.

[35] Zhang X (2022). Three-dimensional electrochromic soft photonic crystals based on MXene-integrated blue phase liquid crystals for bioinspired visible and infrared camouflage. Angew. Chem. Int. Ed..

[36] Hao Q (2018). VO_2_/TiN plasmonic thermochromic smart coatings for room-temperature applications. Adv. Mater..

[37] Gu JX (2021). Fabrication and performances of double-sided HfO_2_ anti-reflection films with ultra-high infrared transmittance. J. Alloy. Compd..

[38] Boyce AM (2022). Actively tunable metasurfaces via plasmonic nanogap cavities with sub-10-nm VO_2_ films. Nano Lett..

[39] Zhao JC (2019). Defining deep-subwavelength-resolution, wide-color-gamut, and large-viewing-angle flexible subtractive colors with an ultrathin asymmetric fabry–perot lossy cavity. Adv. Optical Mater..

[40] Zhao JC (2021). Flexible dynamic structural color based on an ultrathin asymmetric Fabry-Perot cavity with phase-change material for temperature perception. Opt. Express.

[41] Fröch JE (2023). Real time full-color imaging in a Meta-optical fiber endoscope. eLight.

[42] Peng YC, Cui Y (2020). Advanced textiles for personal thermal management and energy. Joule.

[43] Li WL (2022). Dual-color terahertz spatial light modulator for single-pixel imaging. Light Sci. Appl..

[44] Tittl A (2015). A switchable mid-infrared plasmonic perfect absorber with multispectral thermal imaging capability. Adv. Mater..

[45] Song ZY, Chen AP, Zhang JH (2020). Terahertz switching between broadband absorption and narrowband absorption. Opt. Express.

[46] Zeng DW (2022). Dynamically electrical/thermal-tunable perfect absorber for a high-performance terahertz modulation. Opt. Express.

[47] Chen ZG, Segev M (2021). Highlighting photonics: looking into the next decade. eLight.

[48] Ni YB (2022). Computational spectropolarimetry with a tunable liquid crystal metasurface. eLight.

[49] Zhu HZ (2021). Multispectral camouflage for infrared, visible, lasers and microwave with radiative cooling. Nat. Commun..

[50] Chen HH (2019). Graphene-based materials toward microwave and terahertz absorbing stealth technologies. Adv. Optical Mater..

[51] Xu CH (2023). Transient dynamics of the phase transition in VO_2_ revealed by mega-electron-volt ultrafast electron diffraction. Nat. Commun..

[52] Gu JX (2022). VO_2_-based infrared radiation regulator with excellent dynamic thermal management performance. ACS Appl. Mater. Interface.

[53] Kana JBK (2011). Thermally tunable optical constants of vanadium dioxide thin films measured by spectroscopic ellipsometry. Opt. Commun..

