# Supplementary material for: Tunable VO2 cavity enables multispectral manipulation from visible to microwave frequencies
Source: Light Sci Appl. 2024 Feb 21;13:54. doi: 10.1038/s41377-024-01400-w (PMC10879493; doi:10.1038/s41377-024-01400-w)
Supplement: Supplementary file 1 — Supplementary Materials [file 41377_2024_1400_MOESM1_ESM.docx]

Supplementary Information for

**Tunable VO_2_ Cavity Enables Multispectral Manipulation from Visible to Microwave Frequencies**

*Hang Wei ^1, 2^, Jinxin Gu ^3, 4^, Tao Zhao ^1^, Zhiyuan Yan ^2^, He-Xiu Xu ^2^, Shuliang Dou ^1,^***, Cheng-Wei Qiu ^2,^*, Yao Li ^1, 4^**

*^1^ Center for Composite Materials and Structure, Harbin Institute of Technology, 150001, Harbin, China.*

*^2^ National University of Singapore, Department of Electrical & Computer Engineering, Singapore 117583, Singapore.*

*^3^ School of Chemical Engineering and Technology, Harbin Institute of Technology, 150001, Harbin, China.*

*^4^ Suzhou Laboratory, 215123, Suzhou, China.*

Corresponding authors: *[dousl@hit.edu.cn](mailto:dousl@hit.edu.cn);* [*chengwei.qiu@nus.edu.sg*](mailto:chengwei.qiu@nus.edu.sg)*;* [*yaoli@hit.edu.cn*](mailto:yaoli@hit.edu.cn)





**Fig. S1** Roadmap of the current development of multispectral and dynamic manipulation based on PCMs and ECMs. *λ_1_* and *λ_2_* represent the minimum and maximum wavelength of the working region.


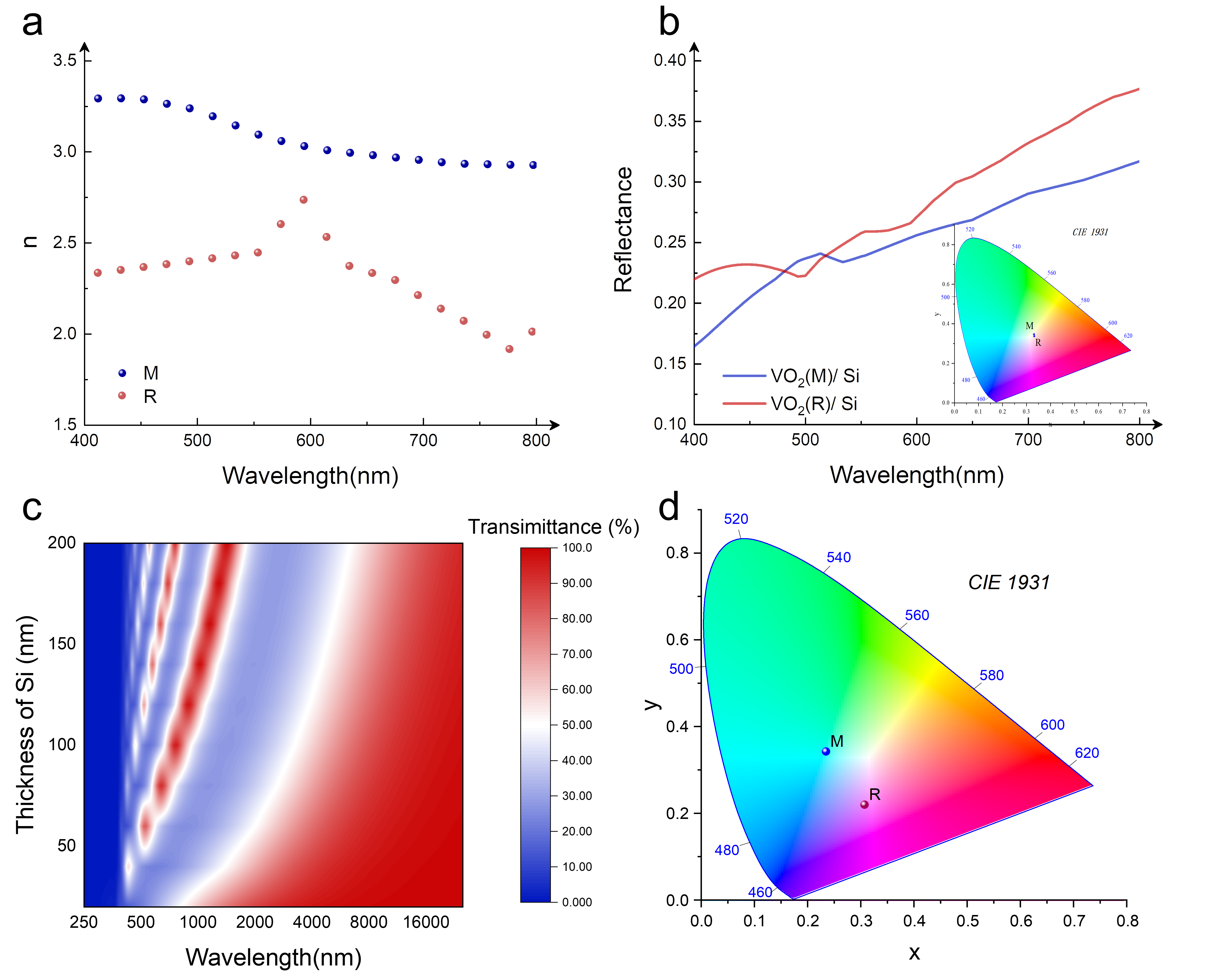


**Fig. S2** (a) Optical constants and (b)visible reflectance spectra of VO_2_ during the phase transition. Attached figure demonstrates surface colors of VO_2_ during the phase transition in CIE 1931 space; (c) The thickness-dependent transmittance spectra of a Si layer in the VIS to IR regions; (d) Simulated color change path across the phase transition of VO_2_ shown in CIE 1930 space when the thicknesses of VO_2_/ HfO_2_/ VO_2_/ Si layers in TFP are 10, 25, 40 and 150 nm.

**Fig. S2a-b** illustrates the color-changing ability of a pure VO_2_ film deposited on a Si substrate. VO_2_ rarely changes its surface color due to the negligible variation of optical constants during the phase transition. Therefore, we propose the TFP structure to amplify the change of optical constants, and further realize the color modification of VO_2_ during its phase transition. **Fig. S2c** demonstrates the transmittance spectra of Si with varying thickness. Considering the blocking effect in the VIS region and the transparency in the IR to MW regions, we choose a 150 nm-thickness Si layer as the reflector of TFP. **Fig. S2d** exhibits the color change shown on the CIE 1931 space for TFP with preset parameters (10nm VO_2_/ 25nm HfO_2_/ 40nm VO_2_/ 150nm Si). TFP shows cyan in the insulating state of VO_2_ while change to purple in the metallic state of VO_2_, which verifies TFP’s capacity to reversibly manipulating the color of VO_2_.


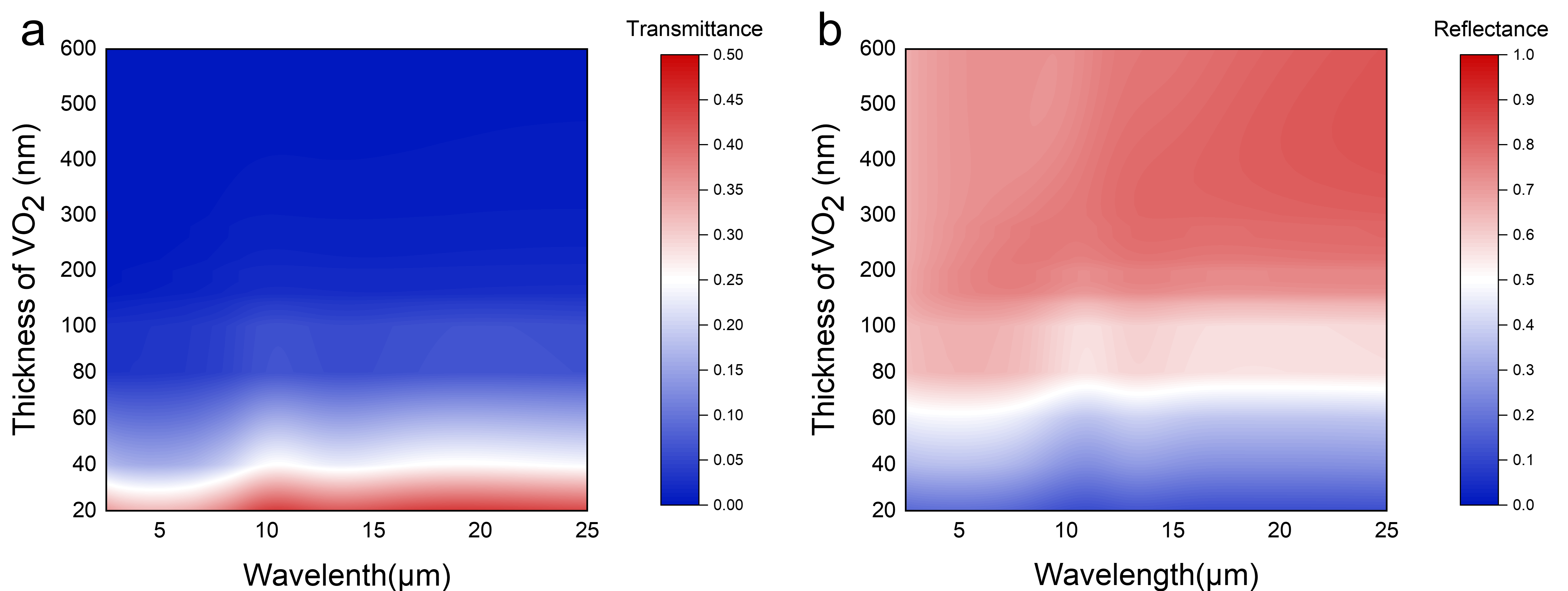


**Fig. S3** Thickness-dependent (a) IR transmittance and (b) reflectance spectra for VO_2_(R).

**Fig. S3a** indicates that a VO_2_(R) layer with a thickness of less than 100 nm can act as a semi-transparent layer when facing the IR waves. When the thickness of VO_2_(R) exceeds 300 nm, IR waves will be completely blocked as a result of the free-carrier plasma resonance of VO_2_(R). Hence, the thickness-dependent optical property enables VO_2_(R) to be able to act as the top semi-transparent layer and the completely reflective mirror in F-P cavities at the same time. Simulations in **Fig. S3** explain the reason why we choose very thin VO_2_ layers in TFP to change the color. If the sum of the thickness of the two VO_2_ layers in TFP exceeds 100 nm, the resonant absorption will almost disappear because most IR waves are reflected by TFP. It is why we emphasize the importance of optical accessibility in all layers.


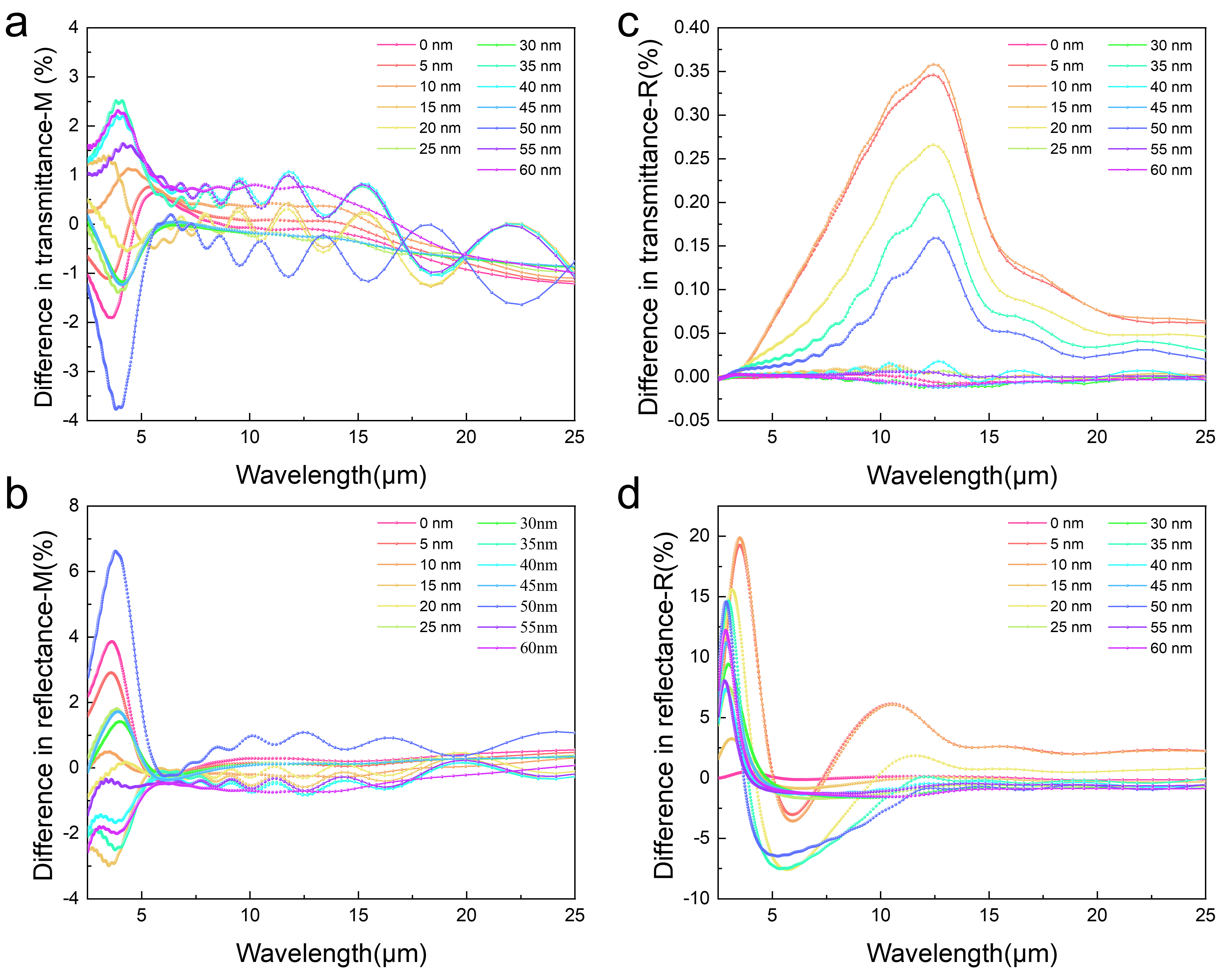


**Fig. S4** Spectral discrepancy in IR between (a) insulating transmittance; (b) insulating reflectance; (c) metallic transmittance; (d) metallic reflectance.

The thicknesses of HfO_2_ and VO_2_ in BFP are initialized to 400 nm and 500 nm, while the thicknesses of HfO_2_ and bottom VO_2_ in TFP are initialized to 25 nm and 20 nm, respectively. Assuming the sum of the thickness of two VO_2_ layers in TFP is *β* ( *β* > 20 nm), we simulated the temperature-dependent IR reflectance and transmittance spectra of two structures: (ⅰ) VO_2_ (*β* - 20 nm)/ HfO_2_ (25 nm)/ VO_2_ (20 nm)/ Si (100 nm)/ HfO_2_ (400 nm)/ VO_2_ (500 nm) and (ⅱ) VO_2_ (*β*)/ Si (100 nm)/ HfO_2_ (400 nm)/ VO_2_ (500 nm). When altering *β* from 20 to 80 nm, we subtract the spectra of the two structures in pairs.

It is clearly observed that the discrepancy between the two spectra is slight, especially in wavelengths longer than 5 μm. In wavelengths shorter than 5 μm, the interfacial effects in TFP, such as the scattering between VO_2_/ HfO_2_, VO_2_/ Si, may affect the spectra. However, due to the sub-wavelength property, the interfacial effect is negligible when the wavelength of electromagnetic waves is further larger than the thickness of layers. Moreover, most IR applications concentrate on a wavelength range of the atmosphere window (8-14 μm), therefore, in the IR region, we can regard the VO_2_/ HfO_2_/ VO_2_ as a monolayer VO_2_, especially in wavelengths longer than 5 μm.


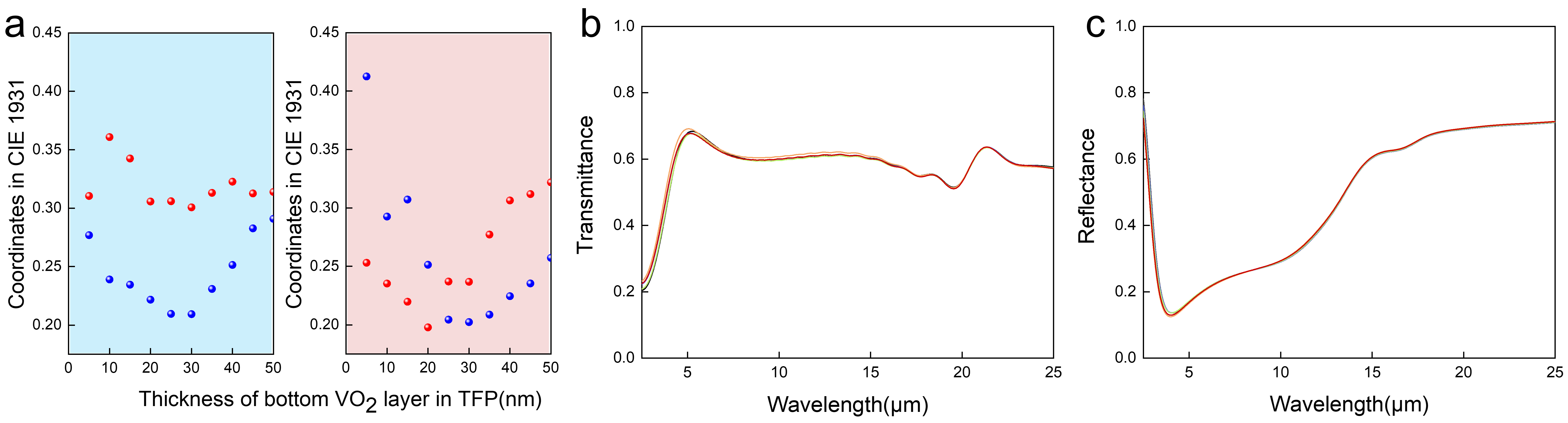


**Fig. S5** (a) The coordinates in CIE 1931 corresponding to different VO_2_ thickness combinations in TFP. The left and right regions represent the insulating and metallic states of VO_2_; (b) IR transmittance (when VO_2_ is insulating) and (c) IR reflectance (when VO_2_ is metallic) spectra of different TFP structures. It should be noted that the sum of the thickness of the two VO_2_ films in TFP keeps 50 nm.

We simulate the VIS colors, IR transmittance and reflectance spectra of different parameters of TFP to verify the wavelength dependence. Thickness of the HfO_2_ layer in TFP is fixed to 25 nm. And the pairs of the thickness of (top VO_2_, bottom VO_2_) in TFP are (0, 50), (5, 45), (10, 40), (15, 45), (20, 30), (25, 45), (30, 20), (35, 45), (40, 10), (45, 5). The results find there is almost no difference between their IR spectra whether VO_2_ is insulating or metallic while the color of system is always changing. It is also strongly confirmed that the IR performance is irrelevant to the thickness combination of the two VO_2_ layers in TFP as long as their sum is fixed. In other words, we can manipulate the surface color-changing paths independently but do not affect the IR or MW spectra.


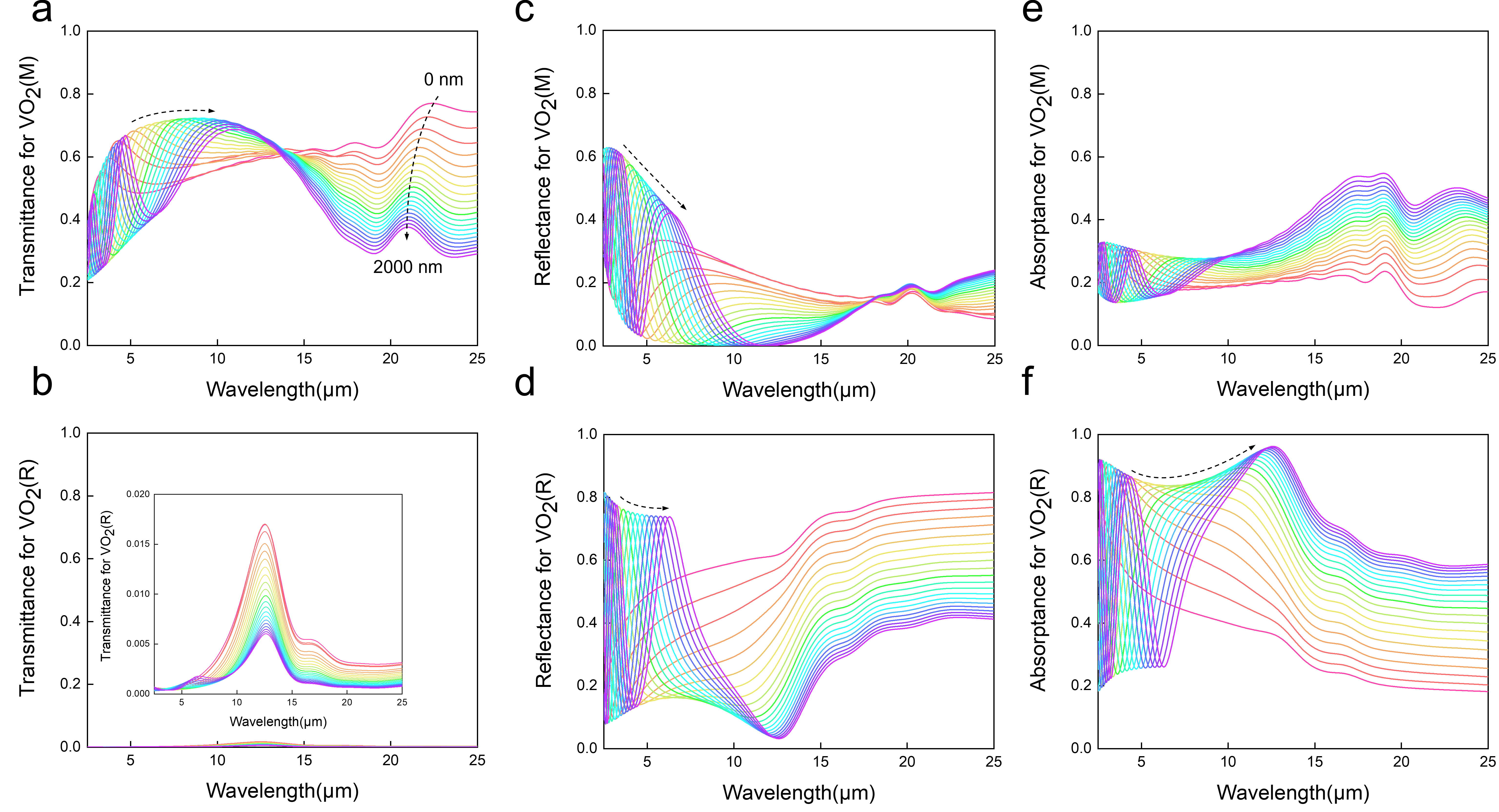


**Fig. S6** Simulated (a) transmittance for VO_2_(M); (b) transmittance for VO_2_(R); (c) reflectance for VO_2_(M); (d) reflectance for VO_2_(R); (e) absorptance for VO_2_(M); (f) absorptance for VO_2_(R) spectra of our system.

We investigate the temperature-dependent transmittance/ reflectance/ absorptance spectra by simulations. When VO_2_ is insulating, as expected, a highly transmissive performance in the IR region can be observed, due to the lossless properties of all layers in most wavelengths. The reflectance and absorptance are therefore low while slightly increased reflectance can be observed in wavelengths shorter than 5 μm. When VO_2_ transforms to the metallic state, the transmittance sharply drops to 0 with increasing reflectance and absorptance. The rising absorptance is induced by the F-P resonance whose peak position is in strong positive correlation with the thickness of HfO_2_ layer in BFP. It is also the appearance of wavelength dependence and endows the ability to alter the peak position within wide ranges according to various applications. In the non-resonant regions, the bottom VO_2_ layer in BFP reflects most IR waves which results in the increasing reflectance.


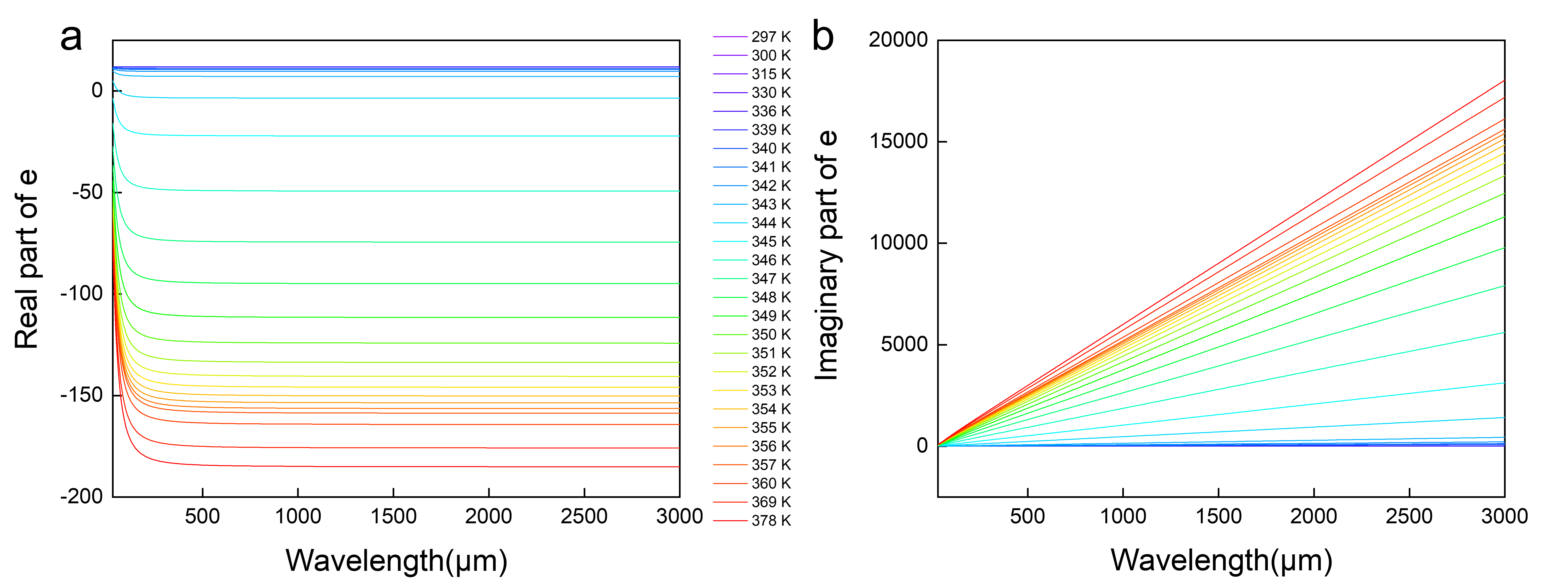


**Fig. S7** Simulated temperature-dependent (a) Real and (b) imaginary parts of the permittivity of VO_2_.

We first measure the temperature-dependent conductivity of a 500 nm-thickness VO_2_ layer deposited on a Si substrate. Then we use the Drude model to calculate the temperature-dependent permittivity of VO_2_ for simulation in the THz and MW regions. it is obviously seen that the VO_2_ layer exhibits a typical semiconductor performance with positive real part of permittivity at low temperatures. When heating the samples to undergo the phase transition, the real part of permittivity decreases fast and becomes negative, indicating the transformation of VO_2_ to exhibit a metallic behavior.


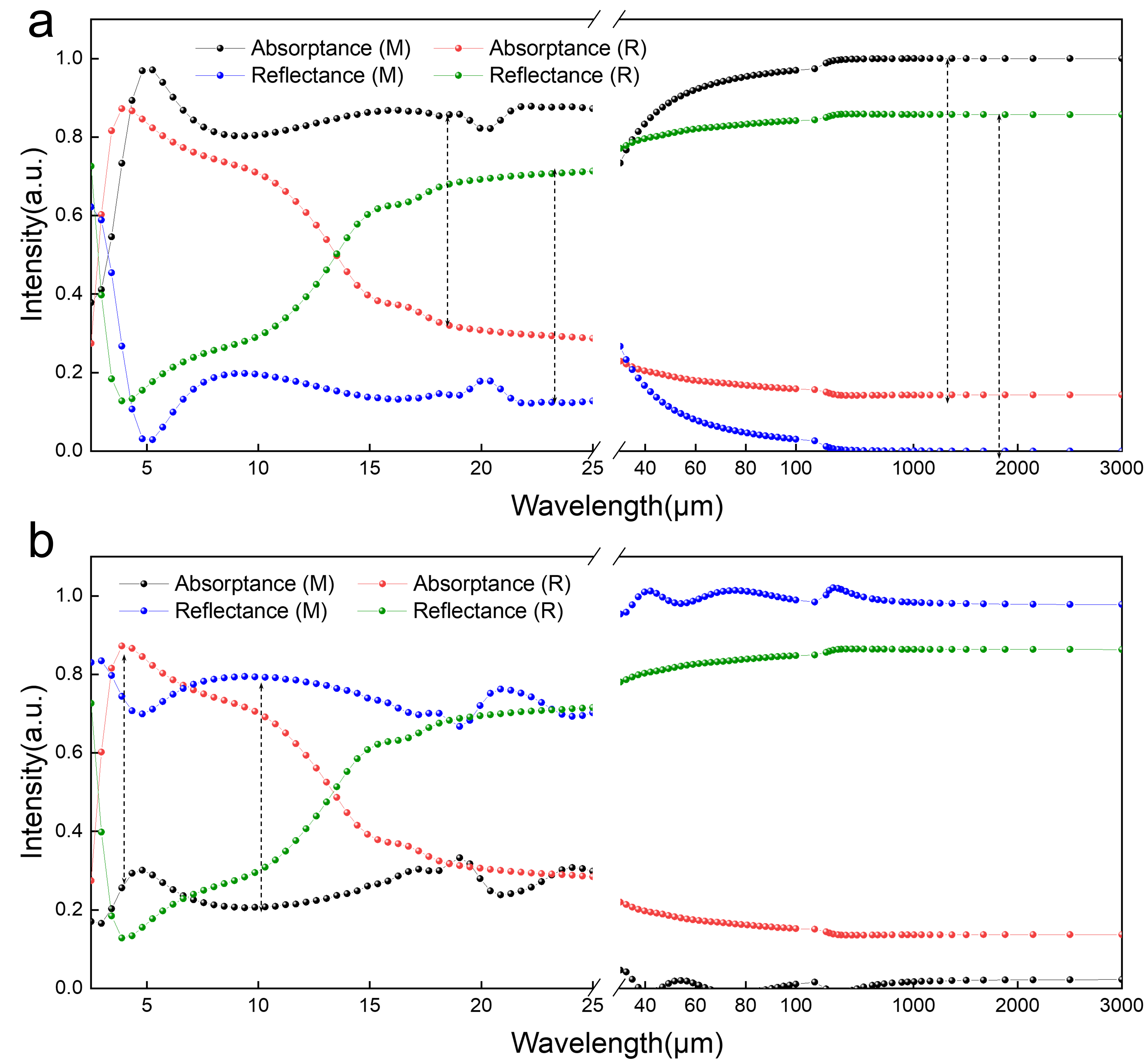


**Fig. S8** Simulated IR-MW spectra of systems deposited on an (a) absorptive and (b) reflective substrate.

We investigate the influence of substrates with different optical properties on the IR to MW regions. The simulated IR to MW spectra in the manuscript can be seen as a result of the system deposited on a totally transmissive substrate, and herein, **Fig. S8** represents the spectra of the system deposited on a completely absorptive and reflective substrate. Due to the blocking effect of the bottom VO_2_ in BFP when it is metallic, the performance rarely changes at high temperatures, while has an apparent difference when VO_2_ is insulating. Therefore, we can choose proper substrates when considering specific applications. For example, if we need to realize multispectral camouflage, we can first choose a substrate with high emittance, so that we can keep the thermal radiation of the device consistent with the surroundings at a wide temperature range. ^[1-2]^ And we can then choose proper color-changing paths by changing the thickness of layers in TFP and customize the absorptance properties at high temperatures by changing HfO_2_ thickness in BFP. For multispectral thermal management, we can choose a substrate with high reflectance. ^[3]^ Transparent substrates are also selective when we consider the applications such as adaptive communications in the MW region or visual concealment. ^[4-5]^


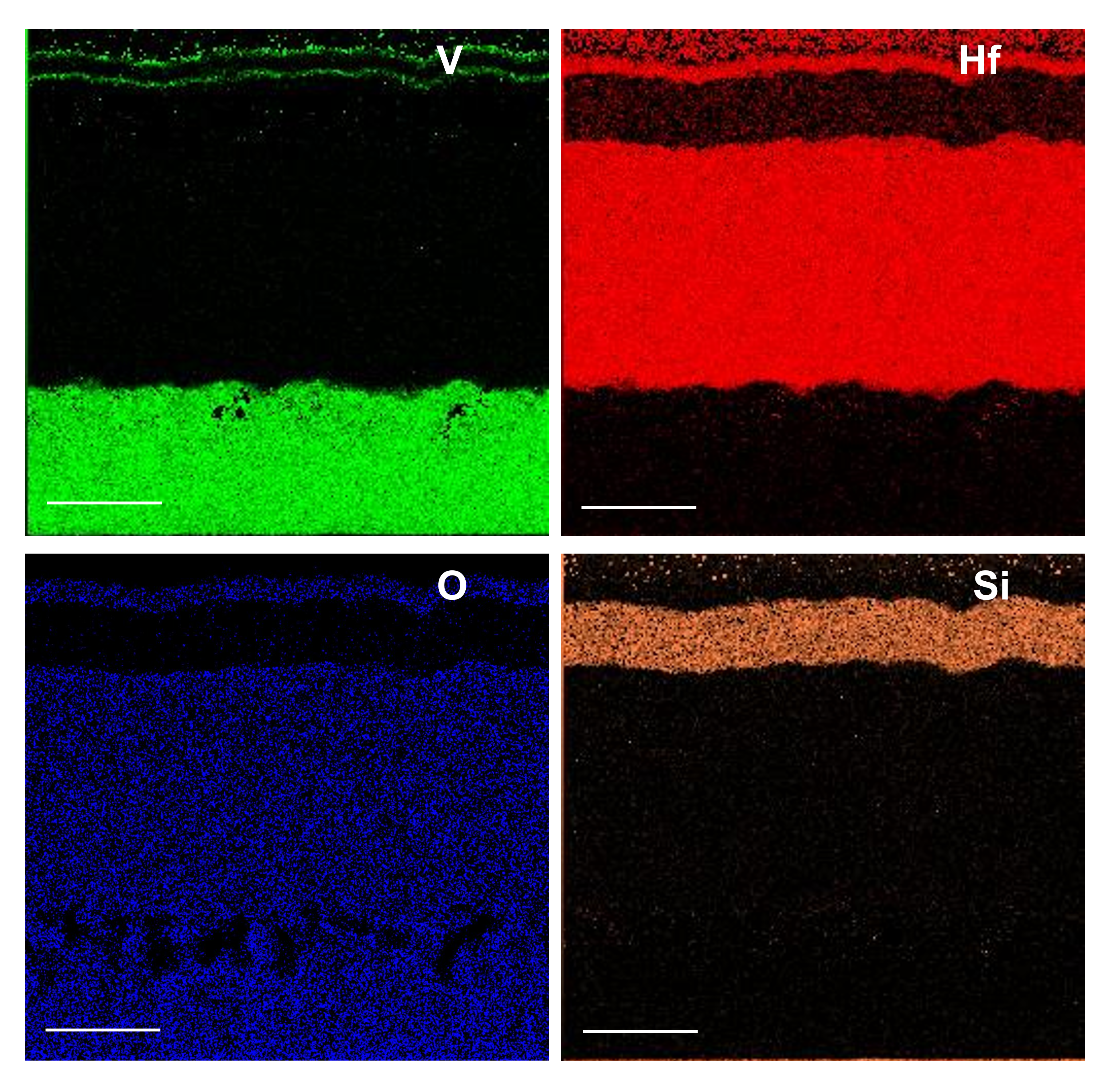


**Fig. S9** EDS mapping images of V, Hf, O and Si elements in cross-sectional HRTEM image of Sample-2. The plotting scale represents 200 nm. The plotting scale in the images represents 200 nm.

It is apparent that the structure consists of six layers of VO_2_/ HfO_2_/ VO_2_/ Si/ HfO_2_/ VO_2_, and the approximate thickness range can be estimated from the mapping images. Some isolated elemental points may be a result of the diffusion of atoms across the interfaces.


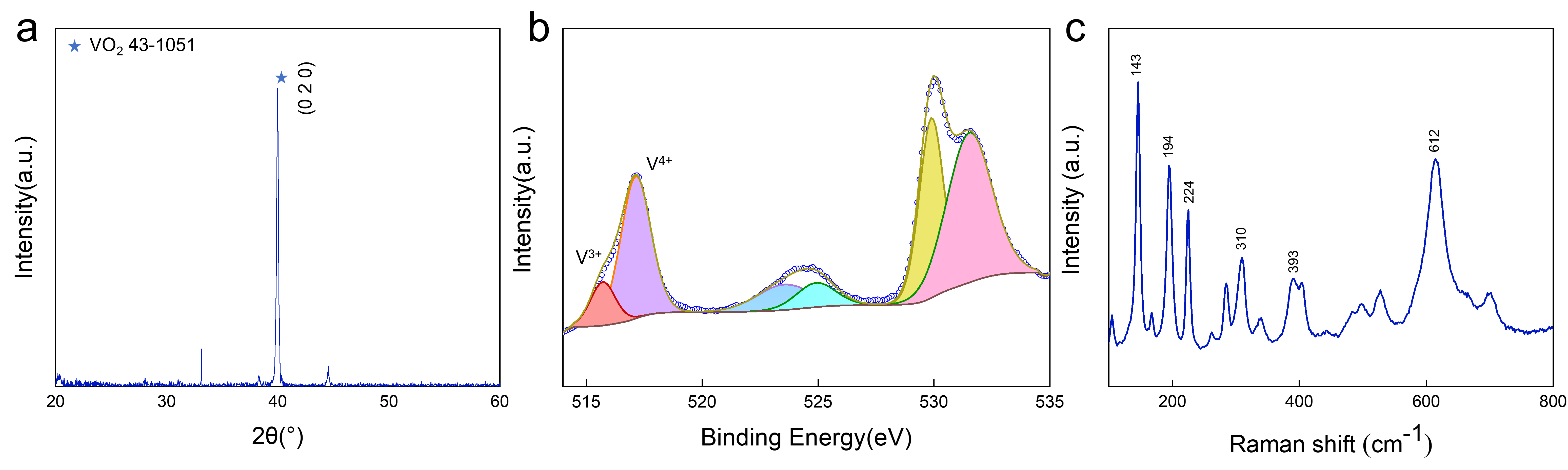


**Fig. S10** Physical properties of the deposited VO_2_ films. (a) XRD patterns; (b) XPS analysis and (c) Raman spectrum.

The sample measured in **Fig. S10** is a 500 nm-thickness VO_2_ deposited on a Si substrate. The XRD pattern indicates the dominant crystal plane of (0 2 0) in the prepared VO_2_ layer according to VO_2_ (JCPDS: 43-1051) with high crystallinity. XPS analysis demonstrates a little position of V^3+^ of V-O composition. The majority of the prepared film is VO_2_ which is verified by the binding energy locating at around 517.1 eV accounting for 2p_2/3_ orbital of V^4+^. Fig 10c exhibits more evidence for a high-quality VO_2_ film. Typical Raman shifts of VO_2_(M) located at 143, 194, 224, 310, 393 and 612 cm^-1^ are observed for its A_g_ and B_g_ phonons. ^[6]^


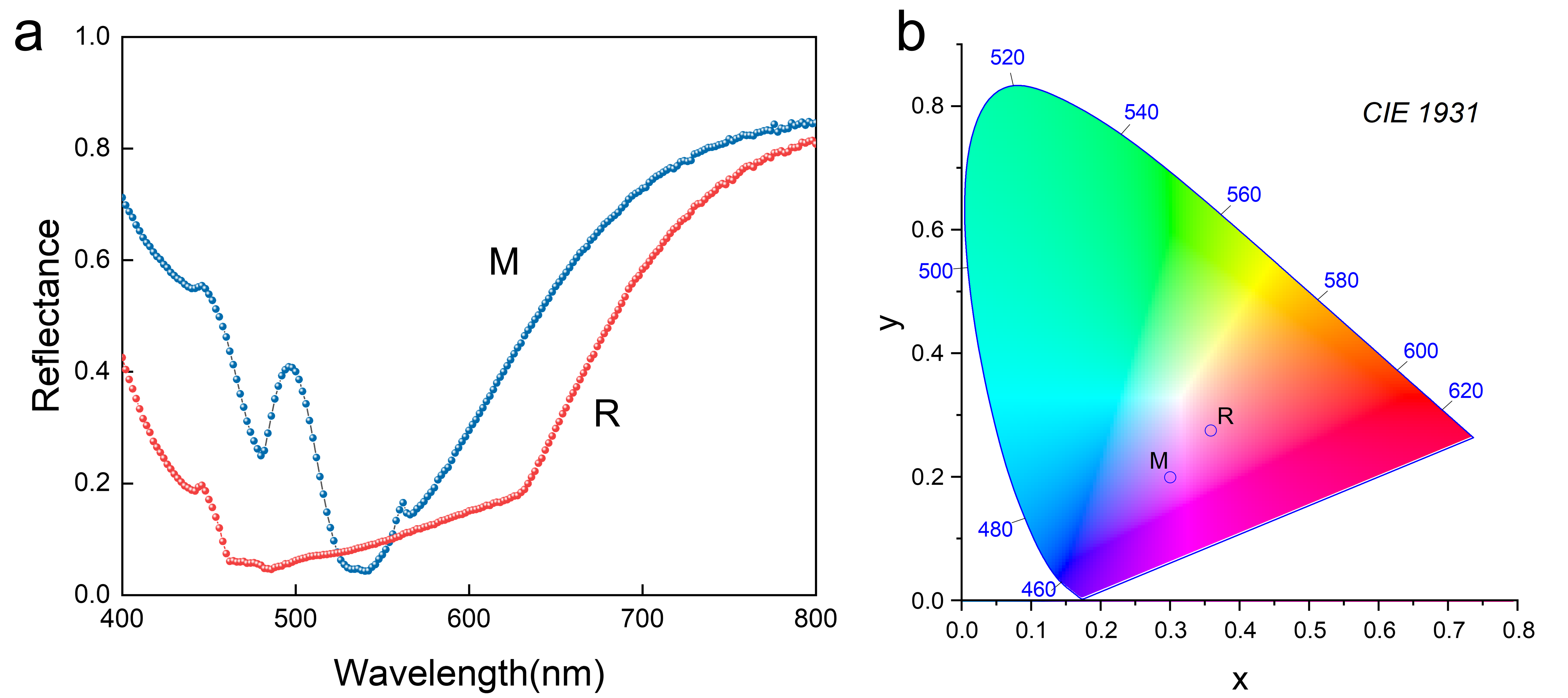


**Fig. S11** (a) Measured temperature-dependent reflectance spectra for PoC samples in the VIS region and (b) corresponding reflective color change in CIE 1931 space.

As shown in **Fig. S11**, measured temperature-dependent VIS reflectance spectra show a very clear blue shift of around 60 nm across the phase transition. The reflectance in non-resonant regions is higher than the simulated reflectance because of some errors between the actual and simulated optical constants. Fortunately, high reflectance in ono-resonant regions will promote the brightness of TFP.


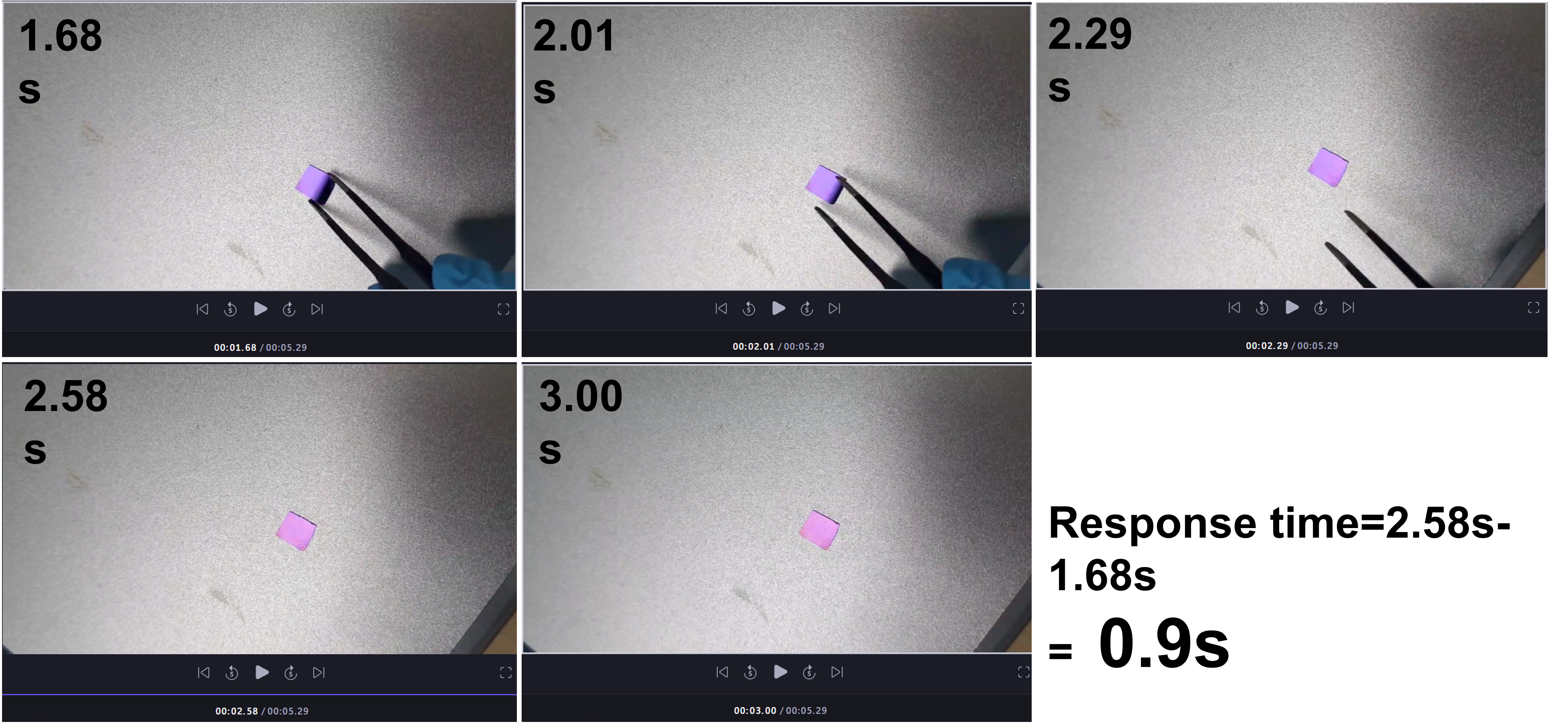


**Fig. S12** The measurement of response time of our system.

We decide to calculate the response time of our system by analyzing the Supplementary Movie.1 frame by frame. As shown in **Fig. S12**, at 1.68 s (the time in the movie), when we put our sample on a 100℃ heater, the surface color of sample changes from purple to pink quickly, and keeps stable at around 2.58 s. Therefore, the response time can be calculated as 0.9 s, which mainly comes from the heat transfer process.^[7]^ It should be noted that the response times for different wavelengths across from the VIS to MW regions are nearly the same, because it is hard to distinguish the intermediate state across the phase transition, when the temperature is high enough to completely trigger its phase transition.


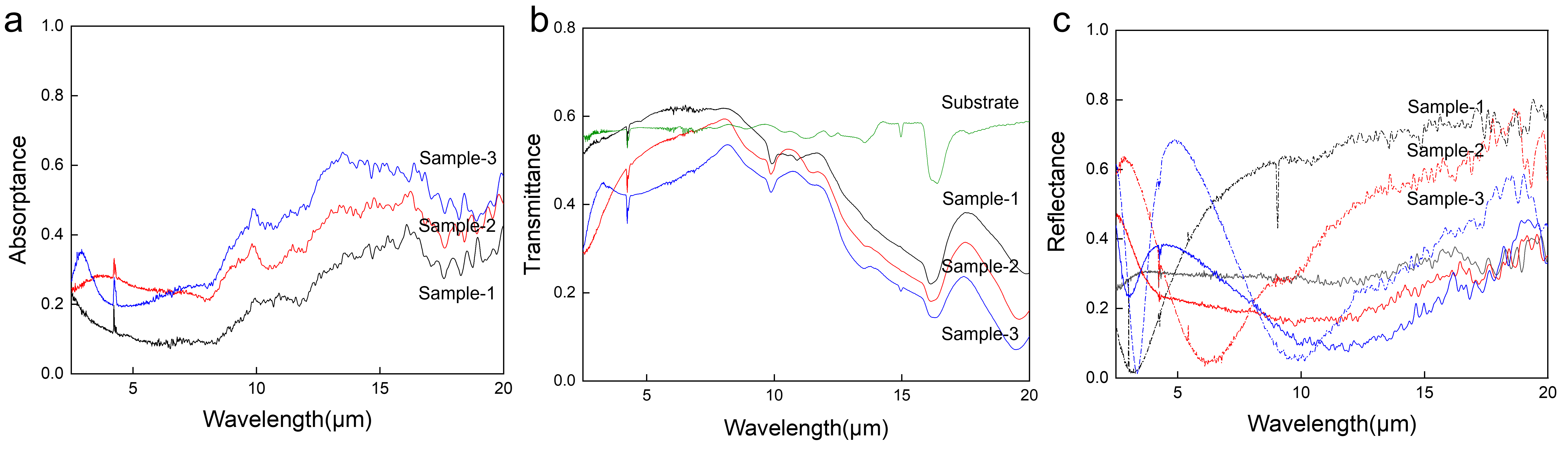


**Fig. S13** Measured (a) absorptance, (b) transmittance and (c) reflectance spectra of PoC samples. Figure a records the absorptance when VO_2_ is insulating. The transmittance when VO_2_ is metallic is all 0 so it is not shown in figure b. The solid and dashed lines in figure c represent the spectra when VO_2_ is insulating and metallic states respectively.

Measured temperature-dependent spectra evaluate the drastic tunability covering transmittance, reflectance and absorptance. It is very important for optical systems with reversible tunability which operate in the IR region, because it is associated with the thermal exchange process. By changing the thickness of the HfO_2_ layer in BFP, the resonant absorption peak can be easily moved within the whole IR range with the peak intensity approaching unity. The absorptance for VO_2_(M) increases with the thickness of HfO_2_ due to its slight absorption, especially at wavelengths longer than 15 μm. ^[8]^ The transmittance for VO_2_(M) is limited to around 0.6 because of the 300 μm-thickness Si substrate. Depositing the multilayers on a substrate with higher transmittance can achieve even promoted tunability of transmittance because it will be zero for a high-temperature transmittance. Meanwhile, aiming at diverse demands in the IR region, the promotion of the tunability of reflectance, especially at longer wavelengths, can be realized by replacing the HfO_2_ dielectric material with lossless materials, such as some metal fluorides. The PoC experiments construct wide platforms for multispectral applications, like multispectral camouflage, thermal management and so on. ^[9-10]^

**Table. S1 Performance comparison of the state-of-the-art optical devices with reversible tunability**

| Category | Ref | Tunable materials | Regions with dynamic response | Wavelength span (λ_2_/λ_1_) | Type of spectral  modulation | External energy input | Customed  spectrum | Response time |
| --- | --- | --- | --- | --- | --- | --- | --- | --- |
| Electrochromic  Devices | [11] | Ag | 2.5-15 μm | 1~10 | R+A | 2.2 V | No | 15 s |
|  | [12] | WO_3_ | 250 - 2500 nm | 10 | R+A | 2.5 V | No | 7.1 s |
|  | [13] | WO_3_/ NiO | 250 - 1000 nm | 1~10 | T+R+A | 2.5 V | No | 30 s |
|  | [14] | Li_4_Ti_5_O_12_ | 400 nm - 13 μm | 10~10^2^ | R+A | 1750 mA·g^-1^ | No | 60 s |
|  | [15] | liquid crystals | 400 nm - 16 μm | 10~10^2^ | R+A | 6 V | No | 70 s |
|  | [16] | liquid crystals | 400 - 500 GHz | 1~10 | T+R+A | 10 V | Yes | 0.4 s |
|  | [17] | Graphene | 400 nm - 3 mm | 10^3^~10^4^ | R+A | 4 V | No | 40 s |
|  | [18] | Graphene | 7 - 12 GHz | 1~10 | R+A | <5 V | Yes | 0.3 s |
|  | [19] | GaAs/Al_0.3_Ga_0.7_As quantum well | ~9.17 μm | ~1 | R+A | 10 V | No | 10^-5^ s |
| Thermochromic  Devices | [20] | liquid crystals | 400 - 1000 nm | 1~10 | T+R+A | 0 | Yes | N/A |
|  | [21] | Ge_2_Sb_2_Te_5_ | 3 - 5 μm | 1~10 | T+R | laser pulse of 60 mJ·cm^-2^ | Yes | ~ ns |
|  | [22] | Ge_3_Sb_2_Te_6_ | 2.5 - 5 μm | 1~10 | R+A | 0 | Yes | N/A |
| Thermochromic  Device | [23] | thermochromic pNIPAm-AEMA microparticles | 250 - 2500 nm | 10 | T+R+A | 0 | No | N/A |
|  | [24] | VO_2_ | 500 nm - 15 μm | 10~10^2^ | R+A | 0 | Yes | N/A |
|  | [25] | VO_2_ | 1 – 3.5 THz | 1~10 | R+A | 0 | Yes | N/A |
|  | **Our work** | **VO_2_** | **400 nm - 3 cm** | **10^4^~10^5^** | **R+A (VIS region)**  **T+R+A (IR to MW regions)** | **0** | **Yes** | **< 0.9 s** |

Note: 1) The wavelength span (λ_2_/λ_1_) represents the wavelengths with tunable optical response under external stimulus, λ_2_ and λ_1_ represent the maximus and minimum of the wavelengths; 2) In the type of spectral modulation, R, A and T represent the reflectance, absorptance and transmittance, respectively; 3) Customed spectrum refers to whether the spectral features (wavelength of absorptance/reflectance/transmittance peak, etc.) can be customed based on the existed design and structure.

**Reference**

[1] Wei, H. et al. Kirigami-Inspired Reconfigurable Thermal Mimetic Device. *Laser & Photonics Reviews* **16**, 2200383 (2022).

[2] Tang, K. C. et al. A Thermal Radiation Modulation Platform by Emissivity Engineering with Graded Metal–Insulator Transition. *Advanced Materials* **32**, 1907071 (2020).

[3] Li, T. et al. A radiative cooling structural material. *Science*. **364**, 760-763 (2019).

[4] Lee, J. et al. Epitaxial VO_2_ thin film-based radio-frequency switches with thermal activation. *Applied Physics Leters.* **111**, 063110 (2017).

[5] Scott, S. et al. A Frequency Selective Surface with Integrated Limiter for Receiver Protection. *2012 IEEE International Symposium on Antennas and Propagation.* **1-2**, (2012).

[6] Shvets, P. et al. A review of Raman spectroscopy of vanadium oxides. *Journal of Raman Spectroscopy* **50**, 1226-1244 (2019).

[7] Gu, J. X. et al. VO_2_-Based Infrared Radiation Regulator with Excellent Dynamic Thermal Management Performance. *ACS Applied Materials Interfaces* **14**, 2683-2690 (2022).

[8] Huang, Y. et al. Hierarchical visible-infrared-microwave scattering surfaces for multispectral camouflage. *Nanophotonics*. **11**, 3613-3622 (2022).

[9] Zhu, H. Z. et al. Multispectral camouflage for infrared, visible, lasers and microwave with radiative cooling. *Nature Communications* **12**, 1805 (2021).

[10] Yang, Z. et al. Oxide Electronics Utilizing Ultrafast Metal-Insulator Transitions. *Annual Review of Materials Research*. **41**, 337-367 (2011).

[11] Li, M. Y. et al. Manipulating metals for adaptive thermal camouflage. *Science Advances* **6**, eaba3494 (2020).

[12] Shao, Z. W. et al. All-Solid-State Proton-Based Tandem Structure Achieving Ultrafast Switching Electrochromic Windows. *Nature Electron*ics **5**, 45-52 (2021).

[13] Li, W. J. et al. Effect of independently controllable electrolyte ion content on the performance of all-solid-state electrochromic devices. *Chemical Engineering Journal* **398**, 125628 (2020).

[14] Mandal, J. et al. Li_4_Ti_5_O_12_: A Visible-to-Infrared Broadband Electrochromic Material for Optical and Thermal Management. *Advanced Functional Materials* **28**, 1802180 (2018).

[15] Zhang, X., et al. Three-Dimensional Electrochromic Soft Photonic Crystals Based on MXene-Integrated Blue Phase Liquid Crystals for Bioinspired Visible and Infrared Camouflage. *Angewandte Chemie* *International Edition* **61**, e202211030 (2022).

[16] Li, W. L. et al. Dual-color terahertz spatial light modulator for single-pixel imaging. *Light: Science & Applications* **11**, 191 (2022).

[17] Ergoktas, M. S. et al. Multispectral Graphene-Based Electro-Optical Surfaces with Reversible Tunability from Visible to Microwave Wavelengths. *Nature Photonics* **15**, 493-498 (2021).

[18] Balci, O. et al. Graphene-enabled electrically switchable radar-absorbing surfaces. *Nature Communictions* **6**, 6628 (2015).

[19] Inoue, T. et al. Realization of dynamic thermal emission control. *Nature Materials* **13**, 928-931 (2014).

[20] McConney, M. E. et al. Thermally induced, multicolored hyper-reflective cholesteric liquid crystals. *Advanced Materials* **23**, 1453-1457 (2011).

[21] Julian, M. N. et al. Reversible optical tuning of GeSbTe phase-change metasurface spectral filters for mid-wave infrared imaging. *Optica*. **7**, 746-754 (2020).

[22] Tittl, A. et al. A Switchable Mid-Infrared Plasmonic Perfect Absorber with Multispectral Thermal Imaging Capability. *Advanced Materials* **27**, 4597-4603 (2015).

[23] Li, X. H. et al. Broadband Light Management with Thermochromic Hydrogel Microparticles for Smart Windows. *Joule*. **3**, 290-302 (2019).

[24] Tang, K. C. et al. Temperature-adaptive radiative coating for all-season household thermal regulation. *Science*. **374**, 1504–1509 (2021).

[25] Zeng, D. W. et al. Dynamically electrical/thermal-tunable perfect absorber for a high-performance terahertz modulation. *Optics Express* **30**, 39736-39746 (2022).
